# The association of HPV-16 seropositivity and natural immunity to reinfection: insights from compartmental models

**DOI:** 10.1186/1471-2334-13-83

**Published:** 2013-02-13

**Authors:** Igor A Korostil, Suzanne M Garland, Matthew G Law, David G Regan

**Affiliations:** 1 The Kirby Institute, University of New South Wales, Sydney, NSW 2052, Australia; 2 Regional World Health Organization Human Papillomavirus Laboratory Network, Department of Microbiology and Infectious Diseases, The Royal Women’s Hospital, 3052, Melbourne, VIC, Australia; 3 Department of Obstetrics and Gynaecology, University of Melbourne, 3052, Melbourne, VIC, Australia; 4 Murdoch Childrens Research Institute, 3052, Melbourne, VIC, Australia

**Keywords:** Seroreactivity, Compartmental model, HPV, Natural immunity

## Abstract

**Background:**

Seroreactivity, processes of seroconversion and seroreversion, in the context of HPV infection has been investigated in numerous studies. However, the data resulting from these studies are usually not accounted for in mathematical transmission models of various HPV types due to gaps in our understanding of the nature of seroreactivity and its implications for HPV natural history.

**Methods:**

In this study we selected a number of simple but plausible compartmental transmission models of HPV-16, differing in assumptions regarding the relation between seropositivity and immunity, and attempted to calibrate them to Australian HPV seroprevalence data for females and males, as well as DNA prevalence data for females, using a Bayesian model comparison procedure. We ranked the models according to both their simplicity and ability to be fitted to the data.

**Results:**

Our results demonstrate that models with seroreversion where seropositivity indicates only a partial or very short-term full protection against re-infection generate age-specific HPV DNA prevalence most consistent with the observed data when compared with other models.

**Conclusions:**

Models supporting the notion that seropositive individuals are fully immune to reinfection demonstrated consistently inferior fits to the data than other models making no such assumption.

## Background

Genital human papillomaviruses (HPV) are viral sexually transmitted infections (STIs) with around 40 types having tropism for the anogenital region. High-risk (oncogenic) HPV types 16 and 18 are more virulent than others and associated with about 70-76% of cervical cancers [1,2]. Of these two, type 16 is more prevalent [3,4] and responsible for the highest proportion of cervical cancers (> 50%) [1,5]. To prevent the spread of cervical cancer and other HPV associated diseases such as genital warts caused by the low-risk (nononcogenic) HPV types 6 and 11, many developed countries are now implementing comprehensive vaccination programs [6-8] utilising highly effective prophylactic HPV vaccines Cervarix® (bivalent vaccine; protects against HPV types 16 and 18) or Gardasil® (quadrivalent vaccine; protects against HPV types 6, 11, 16 and 18).

Because cancer generally develops long after initial infection with HPV, the actual impact of vaccination programs for cancer prevention will not be known for decades after these programs have commenced. Mathematical models have therefore been commonly employed to predict the potential population-level impact of vaccination under different vaccination scenarios and assumptions regarding vaccine properties.

Mathematical transmission models can be constructed in a number of ways but deterministic compartmental models are commonly used due to their relative simplicity and tractability [9-14]. A typical compartmental model is described by a nonlinear system of ordinary differential equations (ODEs) governing changes in the number (or proportions) of individuals in pre-specified subgroups of the modelled population over time.

A necessary element of every modelling study is model calibration. Calibration is performed by adjustment of parameter values to ensure that the model predictions, which are intrinsically uncertain, are consistent with available real-life data. The accuracy of the calibration process can be iteratively improved as more data become available. Often HPV models are calibrated to HPV incidence or prevalence data collected in a particular country or jurisdiction. In view of increasing availability of data related to seroreactivity (production of antibodies in response to infection, known as seroconversion, and their decay, or seroreversion), it is timely to investigate the present possibilities to use them for model calibration. A number of studies (for example, [15-18]) report seroprevalence by age or other characteristics, estimated times from HPV DNA detection to detection of seropositivity, and rates of seroconversion or seroreversion. With the exception of a single modelling study we are aware of [9], these data have not been considered in the development and calibration of transmission because the relationship between seropositivity and immunity is not well understood. However, we believe that it is worthwhile to investigate a few possible associations that may exist between seroreactivity and HPV transmission. The key association that we focus on in this study is that between seropositivity and natural immunity developed after resolution of an HPV infection.

In this study, we develop eight compartmental models based on types SIS (Susceptible-Infected-Susceptible), SIR (Susceptible-Infected-Recovered) and SIRS (Susceptible-Infected-Recovered-Susceptible) [19,20], which incorporate different assumptions regarding the relation between seropositivity and immunity. Our models aim to obey the principle of parsimony, which loosely states that among competing hypotheses the simplest one should be selected. In practice, this means that if the literature presents several conflicting views on an aspect of HPV transmission, we prefer to adopt the one which is described by fewer parameters. By minimizing the number of parameters we increase their explanatory power. We rank the models in terms of their simplicity combined with ability to be fitted to Australian HPV-16 DNA prevalence and seroprevalence data.

## Methods

### Modelled population

Since we intended to calibrate our models to Australian data, it was important to ensure that the population we modelled was a reasonably accurate representation of the sexually active heterosexual Australian population. We defined the modelled population as a set of non-overlapping groups of individuals stratified by gender, age, sexual activity and infection state. In compartmental models (sometimes referred to as population-based models, in contrast with individual-based models), each of these groups (“compartments”) is assumed to be large enough to behave independently of individual stochastic effects. Throughout this paper, when we refer to an “individual” from a particular compartment, we actually mean a descriptor representing the whole population in that compartment, whose attributes are averaged attributes of that population. The age structure of the population was represented by 48 one-year age groups in the range 12 to 59 years of age. This was motivated by the following factors: 1) to model HPV we need to model only the sexually active Australian population, which excludes those younger than a certain age: in our models individuals start sexual activity at 15, but we also included 12–14 year olds, to allow for possible extension of the model should sexual behaviour data for this age group become available; 2) the sexual behaviour data we used [21] do not cover individuals older than 60, and no alternative data were available.

Sexual activity was described by four groups defined by the annual number of new sexual partners. These groups are numbered 1 to 4 in order of increasing activity and contain 60%, 27%, 11% and 2% of the modelled population, respectively [22]. Infection states were as follows: susceptible (S; an individual is susceptible if he or she is at risk of infection); infected (I; an individual who is currently infected; infected individuals are assumed to be both infectious and DNA positive); and recovered (R; an individual in this infection state has resolved his or her infection, tests HPV DNA negative, and is fully immune to re-infection). For compartmental models, the total size of the population is not important for analysis, but was set arbitrarily to 100,000. The population was assumed to be closed, i.e. immigrants, emigrants and temporary visitors were not considered (whether this plays an important role for HPV transmission in Australia has not been established but determining this is outside the scope of the present study). Mortality was also not considered for the following reasons: 1) we did not model progression to cancer and there is no mortality directly associated with HPV infection; 2) the age-specific mortality profile for the age range of the modelled population is fairly flat such that deaths from other causes can be ignored [23]. While births are not modelled explicitly, the modelled population is replenished with 12 year-olds at an annual rate equal to that at which exit from the sexually active population occurs at age 60.

### Sexual mixing

Sexual behaviour in the Australian population is described in our models by means of a mixing matrix which quantifies the rate of new partner acquisition by males and females based on their age and level of sexual activity. Our implementation of the mixing matrix is as previously employed and described in [9,11], and is based on a formulation developed by Garnett and Anderson [24]. The parameters of the matrix are specified as in [22] based on an analysis of data from the Australian Study of Health and Relationships (ASHR) [21].

### Natural history of HPV-16

Differences between the models we evaluate here are in terms of what is assumed in regard to naturally acquired immunity, i.e. immunity acquired as a result of exposure to infection. In this study we evaluate two general scenarios: either individuals cannot become reinfected while being seropositive, or they can. The implementations of seroreactivity, by model, are briefly summarized in Table 1.

**Table 1:** Implementation of seroreactivity in the compared models, their DIC scores and ranking

**Model**	**Effect of seropositivity on transmission**			**Seroconversion before clearance**	**Seroreversion**	**DIC score**	**Rank**
	**No reinfection while seropositive**	**Reduced risk of re-infection while seropositive**	**Risk of re-infection while seropositive is unchanged**				
SIS_1_	–	✓	–	–	–	−101.3	3
SIS_2_	–	✓	–	–	✓	−108.8	1
SIR_1_	✓	–	–	–	–	−28.6	8
SIR_2_	✓	–	–	✓	–	−31.2	7
SIRS_1_	✓	–	–	–	✓	−40.4	6
SIRS_2_	✓	–	–	✓	✓	−45.7	5
SIRS_3_	–	–	✓	–	–	−93.4	4
SIRS_4_	–	–	✓	–	✓	−105.6	2

**Figure 1:**
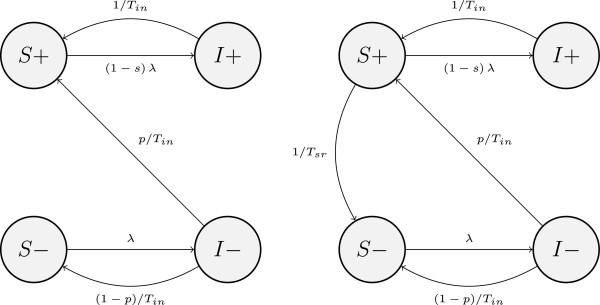
**SIS models: SIS**_**1 **_**(left) and SIS**_**2 **_**(right); “+” and “-” denote seropositivity and seronegativity, respectively.**

The models we refer to as SIS (see Figure 1) are not classical SIS models where individuals become fully susceptible to reinfection following clearance. In our SIS models, individuals enter a model in the S state (susceptibles) being seronegative (hence the state is marked as S-). Then they can acquire a sexual partner who is infected and move to state I (infected) themselves. This is followed by clearance of infection, which can result either in a move back to the state S- (no seroconversion occurred), or to state S+. Individuals in S+ are seropositive and susceptible to reinfection with HPV-16. However, we let them have a degree of immunity varying from none to full (parameter s changing from 0 to 1). This degree is to be inferred by the calibration process. Note that in the limit cases, the models turn into a classical SIR (if s=1), where R is a fully immune state where individuals stay for life, or SIS (if s=0). It is also worth mentioning that the partly immune individuals in S+ are more likely to become infected again if they are more sexually active. This is in contrast to classical interpretations of the immune state usually imposing fixed durations of immunity depending on individuals’ age, but not sexual activity. Model SIS_1_ does not incorporate seroreversion (the decay or loss of antibodies detectable by current assays in an individual): seropositive individuals remain seropositive for life. On the other hand, in SIS_2_ they are assumed to be losing antibodies at a constant rate while in the state S+, so there is a steady migration of individuals from S+ to S- as they lose whatever degree of immunity they had.

In our SIS models females who cleared infection are ensured a degree of natural immunity (see prior distributions in Table 2), while males may or may not be protected.

**Table 2:** Model parameters and their prior distributions

**Parameter description**	**Symbol**	**Prior**	**Source**
Per-partnership probability of transmission from female to male used to calculate the force of infection *λ*.	*β*_*m*_	U(0.10-1.00)	[25]
Per-partnership probability of transmission from male to female used to calculate the force of infection *λ*.	*β*_*f*_	U(0.10-1.00)	[25]
Average duration of infection for males	T_in,m_	U(0.60,1.70)	[26,27]
Average duration of infection for females	T_in,f_	U(0.75,1.50)	[28-30]
Average rate of loss of immunity for males; defined as 1/T_im,m_ i.e. the inverse of the average duration of natural immunity for males	r_li,m_	U(0.01,0.33) (SIRS_1_, SIRS_2_); U(0.01,1.0) (SIRS_3_, SIRS_4_);	[31] (SIRS_1_, SIRS_2_); Not available (SIRS_3_, SIRS_4_);
Average rate of loss of immunity for females; defined as 1/Tim,f i.e. the inverse of the of natural immunity for females	r_li,f_	U(0.01,0.33) (SIRS_1_, SIRS_2_); U(0.01,1.0) (SIRS_3_, SIRS_4_);	[31] (SIRS_1_, SIRS_2_); Not available (SIRS_3_, SIRS_4_);
Probability of seroconversion for males	p_m_	U(0.01,0.30)	[15]
Probability of seroconversion for females	p_f_	U(0.40,0.70)	[32]
Average rate of seroreversion for males	r_sr,m_	U(0.01,0.10)	[33]
Average rate of seroreversion for females	r_sr,f_	U(0.10,1.00)	[18,32,34]
Average degree of immunity for seropositive males	s_m_	U(0.00,1.00)	Not available
Average degree of immunity for seropositive females	s_f_	U(0.10,1.00)	[35-37]
Average time to seroconversion for males (a proportion of T_in,m_)	T_sc,m_	U(0.50,0.95)	[15]
Average time to conversion for females (a proportion of T_in,f_)	T_sc,f_	U(0.50,0.95)	[32,33]
Degree of assortativity by age group	*ε*_*a*_	U(0.10,0.90)	Not available
Degree of assortativity by sexual activity group	*ε*_*r*_	U(0.10,0.90)	Not available

All durations are in years, and all rates are per capita annual rates.

**Figure 2:**
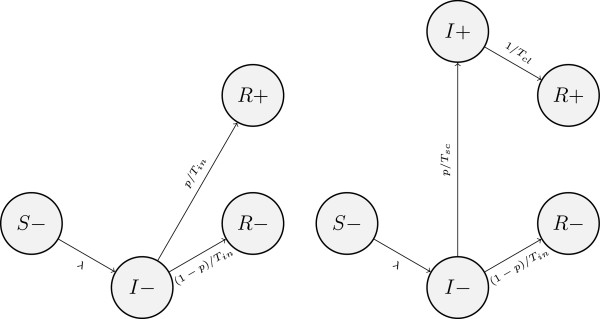
**SIR models: SIR**_**1 **_**(left) and SIR**_**2 **_**(right); “+” and “-” denote seropositivity and seronegativity, respectively.**

The key feature of SIR models (Figure 2) is that infected individuals eventually develop life-long immunity to reinfection. In both models they can clear infection with or without seroconversion. If they do seroconvert, this happens simultaneously with clearance, and seropositive individuals are necessarily in the R state (SIR_1_). In SIR_2_, individuals can test seropositive and still remain infected for some time.

**Figure 3:**
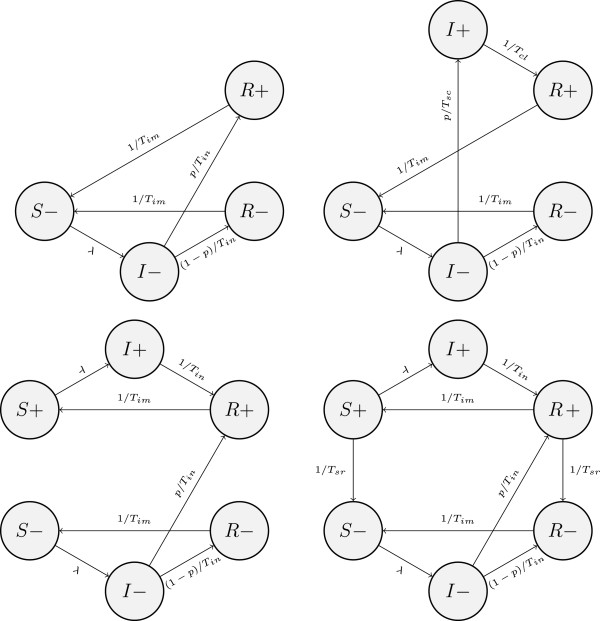
**SIRS models: SIRS**_**1 **_**(top left), SIRS**_**2 **_**(top right), SIRS**_**3 **_**(bottom left) and SIRS**_**4 **_**(bottom right); “+” and “-” denote seropositivity and seronegativity, respectively.**

In SIRS models (Figure 3), where immunity is allowed to wane, individuals return from the R state to the S state.

Model SIRS_1_ is essentially SIR_1_ with waning immunity. Note that we assume no difference in the rates of loss of immunity between seropositive (R+) and seronegative (R-) immune individuals, and losing immunity is equivalent to losing seropositive status. Similarly, SIRS_2_ is an extension of SIR_2_. Model SIRS_3_, just like SIR_1_ or SIRS_1_, assumes that clearance and seroconversion are synchronous, but seropositivity is not an indication of immunity. Consequently, seropositive individuals lose immunity at the same rate as seronegative ones and then can become infected while testing seropositive. Seropositive status is life-long.

Finally, SIRS_4_ is SIRS_3_ with seroreversion. Both susceptible and immune individuals who seroconverted due to previous infection are losing antibodies at a constant rate which is different for males and females.

Note that in order to limit complexity, we chose not to model the scenario whereby an individual can serorevert while infected, since in the infected state the level of antibodies can be assumed to be high - there is, however, no evidence to convincingly support this hypothesis.

Model parameters are gender specific, which allows for possible differences in HPV-16 natural history between females and males. The ordinary differential equations describing the models included in this comparison are provided in the Additional file 1: Technical Appendix.

### Model comparison and calibration

According to the Bayesian approach we adopted, it is necessary to formulate our beliefs about each of the model parameters, before any data produced by the models have been observed, as probability distributions. These distributions are known as prior distributions or “priors”. The priors we used in this study are given in Table 2 and discussed in the Additional file 1: Technical Appendix. We applied a forward simulation procedure with adaptive Markov chain Monte Carlo (as described in [38]) to calibrate each of the models under consideration to Australian HPV-16 seroprevalence data for males and females [17] and DNA prevalence data for females aged 15–39 [39]. During the calibration procedure, quantities required to apply the deviance information criterion (DIC) [40] were calculated. We chose DIC as a means to quantify the parsimony of the models and the goodness of fit of the outcomes they produced to real data after careful consideration of a number of other statistics, such as Bayes factor and Akaike information criterion (see [41,42] or [43] for an extensive overview of approaches to model selection) because it is simple to implement given the samples generated by a Markov chain Monte Carlo simulation. It is pertinent to note that fitting was done to all data at once and the results we obtained would not necessarily coincide with the results produced via fitting to only some of the data (for example, only HPV DNA prevalence). Another important detail is that we did not fix the two sexual mixing parameters *ε*_*a*_ and *ε*_*r*_ describing assortativity by age and sexual activity group (see the Additional file 1: Technical Appendix), instead treating them as ordinary parameters with the assigned uniform prior distributions U(0.1,0.9). This was motivated by the fact that these parameters are very difficult to derive for a particular population based on data from currently available sexual behaviour surveys. Consequently, sexual behaviour was not enforced to be identical in all models.

## Results

The DIC values, calculated for each tested model, are presented in Table 1. There is no rigorous specification of what magnitude of difference in DIC scores indicates a strong preference in favour of a model with the lower score, but some authors recommend using a difference of 10, as a rule of thumb [44]. By this rule of thumb, all models in which seropositivity is associated with full immunity (SIR_1_, SIRS_1_, SIR_2_, SIRS_2_) are clearly inferior to the other models. Allowing seroconversion prior to clearance of infection in SIR_1_, and SIRS_1_ (which turn them into SIR_2_ and SIRS_2_, respectively) somewhat improves their scores, but these are still not competitive. Calibration plots for all models can be found in the Additional file 1: Technical Appendix, along with comprehensive descriptions of the posterior distributions for model parameters.

Here we would like to briefly comment on some of the inferred parameter values for the two “best” models SIS_2_ and SIRS_4_. Firstly, we observe that in SIS_2_ the per-partnership transmission probability from male to female (βf, posterior median 0.806 and the 95% Highest Posterior Density (HPD) interval, i.e. the shortest interval in parameter space which contains 95% of the distribution, (0.514-0.999)) is higher than that from female to male (βm , posterior median 0.59, 95% HPD interval 0.248-0.961)). This is also the case for SIRS_4_, where the posterior mean for βf is 0.885 against 0.695 for βm. These values are consistent with the values predicted in other modelling studies: for example, β (assumed to be the same for female to male and male to female) was estimated at 0.8 (median) with the 95% posterior interval (0.6, 0.99) in [6], at 0.6 in [9] and 0.4 in [7]. There was, however, a study which reported higher rates of female-to-male relative to male-to-female transmission [45]. Posteriors for the average durations of HPV-16 infection are left-skewed for both genders, with median at 1.367 (years) for males and 1.30 for females in SIS_2_ and 1.48 (males) and 1.367 (females) in SIRS_4_. In SIS_2_ the probability of seroconversion for males pm is low (median at 0.135), and for females (pf) it is not higher than the values reported in literature. In particular, its posterior median is at 0.494 while the 95% HPD interval is (0.4-0.654), which is in agreement with 0.5-0.6 suggested in [32]. Somewhat higher pm and pf were observed for SIRS_4_. The inferred values for the degree of immunity for males do not let us make any meaningful conclusions regarding whether or not males are protected, because sm appears to have little influence on the model performance, which is evident from its nearly flat posterior and 95% HPD interval (0.001-0.903). In contrast, the degree of protection for females, sf, has a non-flat posterior, and its 95% HPD interval (0.100-0.810) suggests that we can at least be reasonably confident that it is certainly not complete and does not exceed 0.81, which is an important implication. Another modelling study [14], where degrees of natural immunity were introduced in a manner similar to ours, estimated them at 0.5 for both genders. Rates of loss of immunity (SIRS_4_) were high for both males and females, 95% HPD interval for males is (0.365-0.999) and for females (0.403-1.0). These indicate very short average durations of natural immunity, namely, 1–2.74 years for males and 1–2.48 years for females. Finally, the rates of seroreversion under SIS_2_ are low but higher for females than for males (median 0.08 against 0.03). Under SIRS_4_ these are very similar (median 0.079 for females and 0.03 for males).

## Discussion

The results we obtained show that models assuming that seropositive individuals are fully and permanently protected from reinfection with HPV-16 are clearly inferior to the other models making no such strong assumptions. This conclusion is based on DIC scores. It is important to realise that DIC does not detect a ‘correct’ model in terms of HPV-16 transmission mechanism. Instead, it provides a quantitative model ranking which discourages complexity and is based on the ability of models under consideration (among which the ‘correct’ model may not even be present) to be fitted to the data. Hence, if a simpler model can be calibrated to the data at least as well as a more complex model, it will get a better DIC score. To receive a better DIC ranking, a more complex model would have to justify its complexity by producing a notably better fit than its simpler competitors. To further clarify the context in which our results should be viewed, we mention that our results can be meaningfully interpreted only if we completely rely on the available data – should these be extended or replaced, our results would inevitably change too. Another important aspect is that the DIC ranking factors in how well the models can be fitted to all data at once, for both males and females. If we, for instance, restricted ourselves to only calibrating the models to HPV seroprevalence, the resulting model ranking would likely be different.

As is evident from Table 1, our ‘best’ model is SIS_2_, closely followed by SIRS_4_. The difference in DIC scores between the two models is not substantial and hence does not imply that SIS_2_ is clearly preferable. We should note that the reason why SIS_2_ outscored SIS_1_ is inclusion of seroreversion. Indeed, it is the only difference between the models. Seroreversion in SIS_2_ is implemented with the help of two additional parameters (rsr,m and rsr,f), as compared with SIS_1_, and nonetheless, it improved the fit substantially enough to overcome penalisation for extra parameters and get ahead of SIS_1_ by 8.5 points. The benefits of seroreversion in SIS_1_ are predictable since without it, SIS_1_ can not capture declining seroprevalence in older females. For the same reason, SIRS_4_ provided a significant improvement over SIRS_3_. We see that seroreversion in SIS and SIRS models is crucial in terms of improving the fit to data, even though the rate of seroreversion is low.

Although the highest ranking models SIS_2_ and SIRS_4_ have different structures, as we mentioned in Results, the fitted durations of full natural immunity in SIRS_4_ are very short. Hence, this model is approaching a limit case when it almost becomes SIS_2_ (see Figure 1 and Figure 3).

It is our view, given what is currently known about immunity (in particular, the reported association between seropositivity and reductions in the number of incident infections in seropositive individuals [34,35]), that the protection mechanism assumed in SIS_2_ may be a more realistic representation of naturally acquired protective immunity than a short but full immunity as in SIRS_4_.

It is important to note that nearly all information available regarding the possible association of seropositivity with protective immunity has come from studies of females. The only study of males in this context that we are aware of [16] suggests that for males seropositivity is possible without any immunity. No substantiated inferences in regard to the existence of protective immunity in males resulted from our study: SIS_2_ was not sensitive to variations in the degree of immunity in males. To increase sensitivity, the amount of data used for model specification and calibration and/or their accuracy should be increased, which we expect to happen in future, when, for example, HPV DNA seroprevalence data for males become available.

Our models have a number of limitations. In particular, we assumed the duration of immunity to be the same for all ages, which is unlikely to be true in reality, and the probability of seroconversion to be independent of an individual’s age though there is some evidence to the contrary [34]. Additionally, compartmental models are inherently biased in certain respects. Because they assume a sexual contact is effectively instantaneous, to achieve better fit to real data, compartmental models need to compensate for a somewhat lowered level of sexual activity by maintaining higher probabilities of transmission and longer durations of infection (see [46] for detailed discussion). It is also important to remember that considerable uncertainty remains in our understanding of HPV natural history which influence our specification of priors for model parameters. Also, reliability of data obtained from sexual behavior surveys may be arguable. Finally, the results of this study rely on the data we calibrated our models to, which had their own limitations (see [17,39] for discussion). Perhaps, the most evident limitation is that HPV-16 prevalence data only covered women aged 15–39.

## Conclusions

In conclusion, the models which provided the optimal combination of parsimony and goodness of fit to the currently available Australian data are these where seropositivity indicates only a partial (or very short full) immunity against re-infection and seroreversion is assumed to be taking place. Future studies will no doubt provide greater insight into the nature of acquired immunity and its association with seropositivity, enabling us to build more accurate models.

## Competing interests

The authors of this manuscript do not perceive any direct conflict of interest in relation to the research described. However, in the interest of full disclosure, we declare the following:

The research described in this manuscript was funded by an Australian Research Council Linkage Project (LP0883831). CSL Limited, the distributor of Gardasil® in Australia and New Zealand, is a Partner Organization on this project.

Igor Korostil’s salary is funded by the above mentioned grant (LP0883831).

Professor Suzanne Garland has received advisory board fees and grant support from CSL Ltd and GlaxoSmithKline (GSK), and lecture fees from Merck, GSK and Sanofi Pasteur; in addition, she has received funding through her institution to conduct HPV vaccine studies for Merck and GSK. She is a member of the Merck Global Advisory Board as well as the Merck Scientific Advisory Committee for HPV.

Dr David Regan has received honoraria from CSL Ltd for advisory board participation and for presenting his work at sponsored symposia. He has also received grant support from CSL Ltd for other HPV-related research projects.

## Authors' contribution

MGL and SMG participated in study design, and manuscript preparation. DGR coordinated the study and participated in study design, model parameterisation and manuscript preparation. IAK conceived of the study, developed and implemented the models, analysed results and drafted the manuscript. All authors read and approved the final manuscript.

## Pre-publication history

The pre-publication history for this paper can be accessed here:

http://www.biomedcentral.com/1471-2334/13/83/prepub

## Supplementary Material

Additional file 1 Technical Appendix. Click here for file
