# Supplementary material for: The association of HPV-16 seropositivity and natural immunity to reinfection: insights from compartmental models
Source: BMC Infect Dis. 2013 Feb 13;13:83. doi: 10.1186/1471-2334-13-83 (PMC3599087; doi:10.1186/1471-2334-13-83)
Supplement: Additional file 1 — Technical Appendix. [file 1471-2334-13-83-S1.doc]

**Technical Appendix**

**Igor A. Korostil^1^, Suzanne M. Garland^2, 3, 4^, Matthew G. Law^1^ and David G. Regan^1^**

^1^The Kirby Institute, University of New South Wales, Sydney, NSW 2052, Australia

^2^Regional World Health Organization Human Papillomavirus Laboratory Network, Department of Microbiology and Infectious Diseases, The Royal Women’s Hospital, Melbourne, VIC 3052, Australia

^3^Department of Obstetrics and Gynaecology, University of Melbourne, VIC 3052, Australia

^4^Murdoch Childrens Research Institute, VIC 3052, Australia

## Model equations

All equations use brackets to denote the number of individuals in a state. Each state is stratified by gender, sexual activity group and age group (not shown explicitly to simplify notations). Equations are solved sequentially on time intervals [0,1] which correspond to 1 year. An initial condition imposed at the beginning of each time interval is that the number of individuals of age
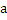
 in each state is the number of individuals aged
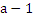
 calculated at the end of the previous interval (this way we implement the process of aging). The exception to this is the first age group, to which we simply add new susceptible individuals at the beginning of each interval.

### Model SIS_1_


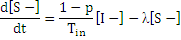


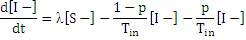


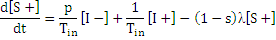


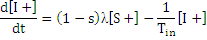


### Model SIS_2_


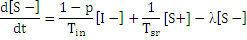


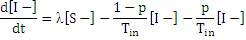


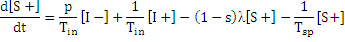


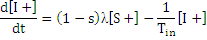


### Model SIR_1_


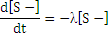


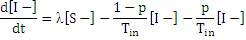


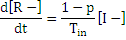


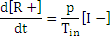


### Model SIR_2_


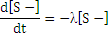


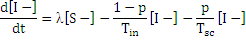


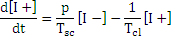


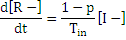


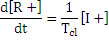


### Model SIRS_1_


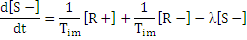


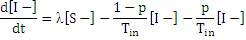


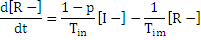


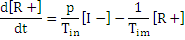


### Model SIRS_2_


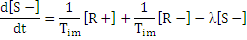


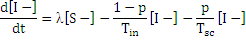


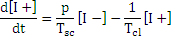


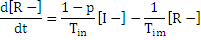


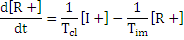


### Model SIRS_3_


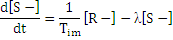


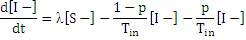


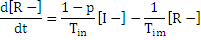


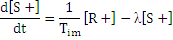


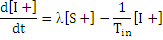


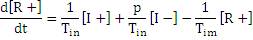


### Model SIRS_4_


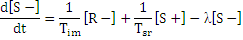


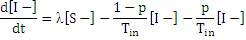


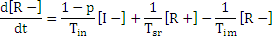


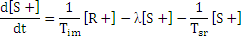


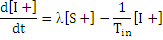


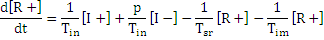


## Calculation of the force of infection

Force of infection, usually denoted
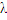
, is a rate at which an individual becomes infected per unit time. In our models it is a yearly rate. For example, the force of infection on females of a given age
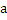
 and sexual activity level
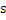
 is defined as

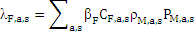


where
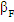
 is a probability of HPV transmission from male to female per partnership,
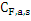
 is a mean partner change rate for females of this category,
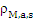
 is a probability that these females get male partners of a particular age and sexual activity who are infected with probability
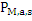
 (which is assumed to be equal to HPV prevalence in males of the category in question). Note that HVP prevalence is the proportion of all (both seropositive and seronegative) individuals testing DNA positive.

In this paper we use the implementation of sexual mixing developed by Garnett and Anderson . The sexual behavior data required for this implementation (originally presented in and derived based on the ASHR survey results ) are as below.

| Sexual activity group | 1 | 2 | 3 | 4 |
| --- | --- | --- | --- | --- |
| Percent of population in the group | 60 | 27 | 11 | 2 |
| Relative yearly partner change rate | 1 | 4.76 | 24.83 | 105.65 |

| Age group | 16-19 | 20-24 | 25-29 | 30-34 | 35-39 | 40-44 | 45-59 |
| --- | --- | --- | --- | --- | --- | --- | --- |
| Sexual partner change rate, per year | 5.28 | 6.06 | 4.37 | 2.57 | 1.61 | 1.43 | 1 |

## The overall annual sexual partner change rate for the entire Australian sexually active population was fixed at 0.43.

## Selection of prior distributions

**Per-partnership probability of HPV-16 transmission from female to male,**
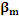
 and from male to female,
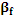


An estimation of this probability at 0.20 (95% CI, 0.16–0.24) was reported by the HITCH Study (HPV Infection and Transmission among Couples through Heterosexual activity) [25]. The study enrolled 18-24 y.o. women and their partners, 179 discordant couples in total. No notable differences were detected between the probabilities female-to-male and male-to-female transmissions.

However, taking into account a considerable uncertainty surrounding this probability, especially, in the context of compartmental models where all partnerships are treated as instantaneous, we decided to use the reported estimation to define only the lower boundary of the prior, that is, we have assigned a uniform distribution U(0.1,1.0) to both
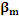
 and
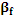
.

**Average infection for females,**
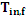


We selected as our primary source the extensive Ludwig-McGill cohort study [30] which recruited 2,462 women from Brazil aged 18-60. The study reported mean duration of 11.9 (10.3–13.5) (or in years 0.99 (0.86-1.12)) and median 7.3 (6.3–10.7) (in years 0.6 (0.52-0.9)).

A cohort of 331 women aged 18-35 years was examined within the Young Women’s Health Study in the USA [28]. The median duration of infection was found to be 9.8 months (0.81 years).

On the other hand, a Canadian study which recruited 635 female university students attending either the McGill or the Concordia University Health Clinic [29] reported notably longer mean duration of infection of 18.3 months (95% CI: 12.9-23.7), or 1.52 (1.07-1.97) in years.

Given this information, we selected a uniform prior distribution U(0.75,1.5) for this parameter.

**Average duration of infection for males,**
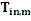


A study conducted in Tucson, USA covered 290 men aged 18 – 44 years and reported a median duration of 6 months (95% CI: 5.2-6.8), which is in years 0.5 (0.43-0.57) [27]. The HPV in Men (HIM) study (1159 men aged 18-70 years from USA, Brazil and Mexico) derived the median duration of 12.19 months (95% CI: 7.16-18.17) or 1.01 (0.6-1.56) in years [26].

To select a prior for the mean duration of infection we assumed that just like for females, the mean is likely to be greater than median, and selected U(0.6,1.7) as a prior.

**Average rate of loss of natural immunity for males,**
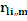
 and **females,**
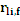


We distinguish between two cases here. One is when seropositivity indicates full immunity (models SIRS_1_  and SIRS_2_ ). In these models an individual who is seropositive can not become re-infected, so the loss of immunity is simultaneous with the loss of seropositivity. This implies that though we have no data on the duration of immunity, we do have some on the duration of seropositivity which we can use. In particular, the reported minimal durations of seropositivity vary from 3 to 7-13 years [31], which lets us assume the minimal duration of natural immunity to be 3 years. For males, we assumed the same minimal durations by analogy.

The second case is when seropositivity is not limited to the immune state: an individual can become susceptible or infected while still remaining seropositive (models SIRS_3_  and SIRS_4_ ). Then there is no reason to restrict the minimal duration of immunity to at least 3 years, so we assumed it is equally likely to be from 1 year to 100 years.

In our models the actual parameter we used was the rate of loss of immunity, defined as 1/
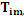
. Given the considerations above, this rate was uniformly distributed according to U(0.01,0.33) for case one, and U(0.01,1.00) for case two.

**Probability of seroconversion for males,**
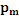


A recent study covering 18-21 y.o. male students (156 in total) recruited at the University of Washington in Seattle, at 2 years from first detection of genital HPV infection percentage of seroconverted was estimated at 13.0 (95% CI: 6.6-24.8).

Based on this, we selected a uniform prior U(0.01,0.3).

**Probability of seroconversion for females,**
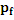


Females university students aged 18-20 were studied in . About 50% of them seroconverted at 1 year after the first HPV-16 DNA detection, and at 2 years the percentage increased to about 60%,. Then it stayed at approximately the same level.

In view of this, we selected a uniform prior distribution U(0.4,0.7) for this parameter.

**Rate of seroreversion for males,**
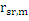


A study covering 809 females and 768 males over 14 was performed within the project RESPECT [33]. At baseline, 147 out of 768 males were seropositive, and at 1 year of follow up, the number of seropositive males decreased to 139, which is a 5.44% reduction.

Hence, we selected a wide uniform prior U(0.01,0.1), i.e. 1% to 10%.

**Rate of seroreversion for females,**
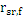


The Finnish Family HPV Study reported antibody decay in 290 women (mean age 25.5 years). During the median follow-up time (37.2 months), decay of antibodies to HPV 16 was observed in 5.3% of women.

Females university students aged 18-20 residing in the state of Washington were studied in . It was observed that 20 (71.4%) of 28 women HPV-16 seropositive at all visits (the average length of follow-up was 31.3 months).

A study conducted in Guanacaste, Costa Rica reported that 55% (675 of 1216) of women seropositive at enrolment (1993-94) remained seropositive for HPV-16 at follow-up (2000).

To ensure that these somewhat conflicting data would be taken into account in our comparison, we chose a uniform prior distribution U(0.01,0.5).

**Degree of natural immunity for seropositive males,**
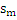


Since no data were available to inform the choice of a prior distributions for this parameter, we selected U(0.0,1.0), that is, the degree was assumed to be equally likely anywhere in the range from 0 (non-existent) to 1 (full immunity).

**Degree of natural immunity for seropositive females,**
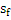


A total of 151 incident HPV16 infections were observed in a cohort of 974 women from the Guanacaste Natural History Study . Both VLP ELISA and cLIA were associated with protection against subsequent HPV infections. Although the point estimate of the cLIA result showed stronger protection, the difference was not significant (HPV16 cLIA: OR, 0.44 [95% CI, 0.21–0.93]; HPV16 VLP ELISA: OR, 0.56 [95% CI, 0.33–0.93]; sampling-adjusted estimate for HPV16 cLIA: OR, 0.37 [95% CI, 0.15–0.94]; sampling- adjusted estimate for HPV16 VLP ELISA: OR, 0.54 [95% CI, 0.29–1.03]).

A study based at Rutgers University [36] recruited 608 female students (mean age 20) and concluded that for the subjects who had persistently high levels of IgG to HPV-16 VLPs for >2 visits, their relative risk for subsequent infection was close to zero.

A Costa Rican study [37] with women aged 18–25 years at enrollment observed that having high HPV16 antibody titer at enrollment was associated with a reduced risk of subsequent HPV16 infection (women in the highest tertile of HPV16 antibody titers, adjusted rate ratio 0.50, 95% CI 0.26-0.86 vs HPV16-seronegative women).

This information can be interpreted as an indication that there is a nonzero degree of immunity for females, which is likely to be significant. To make sure that the reasonably widest range of possible values is covered, we chose a uniform distribution U(0.1,1.0).

**Average time to seroconversion for females,**
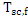


A study covering 809 females and 768 males over 14 was performed within the project RESPECT [33]. Of 229 cases of incident HPV-16 seropositivity, 137 (59.8%) had seroconverted by the 6-month follow-up visit and 92 (40.2%) had seroconverted by the 12-month follow-up visit.

Females university students aged 18-20 residing in the state of Washington were studied in . For 42 subjects under observation, the median time to seroconversion from DNA detection was 11.8 months (0.98 years).

This parameter was actually implemented as a fraction of the average duration of infection, which appears to be at least half of it. Hence we used a uniform distribution U(0.5,0.95).

**Average time to seroconversion for males,**
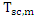


Based on the results of a recent study covering 18-21 y.o. male students (156 in total), we, just like for females, implemented this time as a fraction of the average duration of infection drawn from a uniform distribution U(0.5,0.95).

**Average time to clear infection following seroconversion for females or males,**
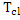


This time is not a separate parameter in the models (SIR_2_ and SIRS_2_), it is only used as a notation. In fact, it is equal to the average duration of infection minus the average time to seroconversion.

**Sexual mixing parameters**

All our models had two sexual mixing parameters: degrees of assortativity by age (
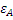
) and sexual activity group (
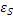
). These were assigned the following prior uniform distributions:
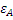
~U(0.1,0.9) and
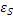
~U(0.1,0.9).

## Data used to calibrate the models

**Seroprevalence data**

We used the age-specific seroprevalence data reported in . While the detailed discussion on the data and methods of its collection are provided in the paper we refer to, here we briefly mention some of that information.

To collect serum samples, public and private laboratories from New South Wales, Victoria, and Queensland were contacted. These 3 states account for about 80% of Australian population. Recorded demographic data were the following: age group, sex, and date of sample collection. The age groups were specified as 0–4, 5–9, 10–14, 15–19, 20–29, 30–39, 40–49, 50–59, and 60–69 years of age.

The sample size was calculated taking into account the mean cumulative number of lifetime sexual partners by age cohort, as reported by the Australian Study of Health and Relationships which we use in our study. The number of samples from females was 1,523 and from males 1,247.

The samples were tested at Merck Research Laboratories (Wayne, Pennsylvania). The overall population HPV seroprevalence was estimated via weighting to Australian population estimates by age.

## HPV DNA prevalence data for females

The seroprevalence data we employed were from the recent the Women’s HPV Indigenous Non-Indigenous Urban Rural Study (WHINURS) study as reported in [39]. The study covered 655 Indigenous and 1,494 non-Indigenous women aged 18 to 40 years who were attending their usual healthcare provider for routine Pap smear cytology . Women represented all Australian states and one territory (Northern Territory) of Australia and the data were obtained from 16 Indigenous health services, 8 family-planning services and 10 community clinics.

Age-adjusted prevalences were calculated by weighting the WHINURS sample to the relevant Australian Bureau of Statistics (ABS) population structure, by single year of age.

Among the key limitations, apart from not inclusion of women over 40, are that the sample was not geographically or demographically representative of all Australian women and non-Indigenous women who attended free community health services, were often likely to be of lower socioeconomic status on average than other Australian women (see [39] for discussion).

**MCMC trace plots, posterior density plots, quantitative summaries of posterior distributions and calibration plots for all models**

We obtained 120,000 samples from posterior distributions for each model parameter. MCMC trace plots, posterior density plots, posterior means, medians, etc. were produced using the R package CODA (Output analysis and diagnostics for Markov Chain Monte Carlo simulations).

Calibration plots show the actual data (means) with 95% confidence intervals (gray whiskers) as well as the simulated means with 0.975 and 0.025 quantiles over the last 100,000 of MCMC samples.

Finally, the 95% Highest Posterior Density (HPD) interval presented here is the shortest interval in parameter space which contains 95% of the distribution.

**Model SIS_1_**


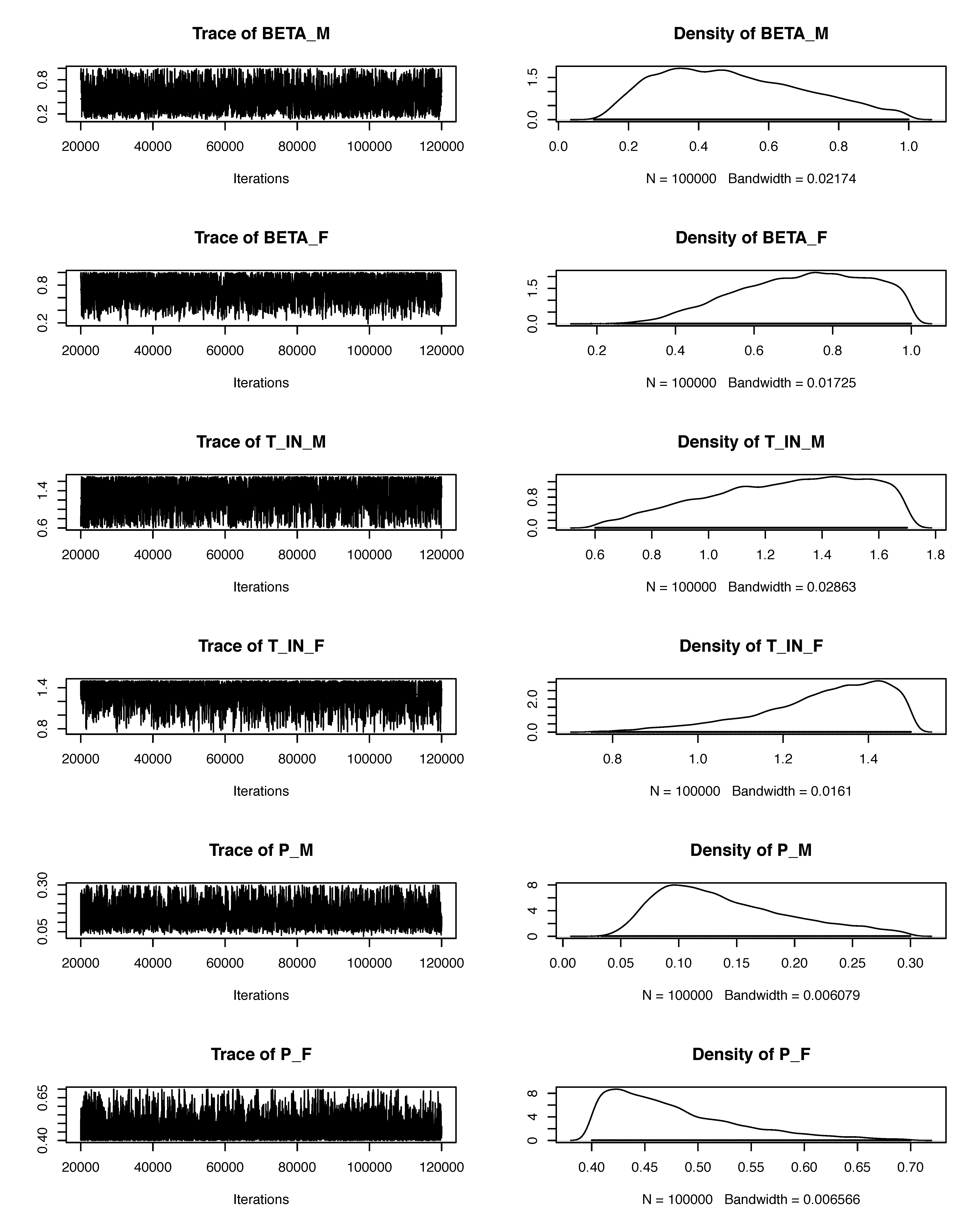


**
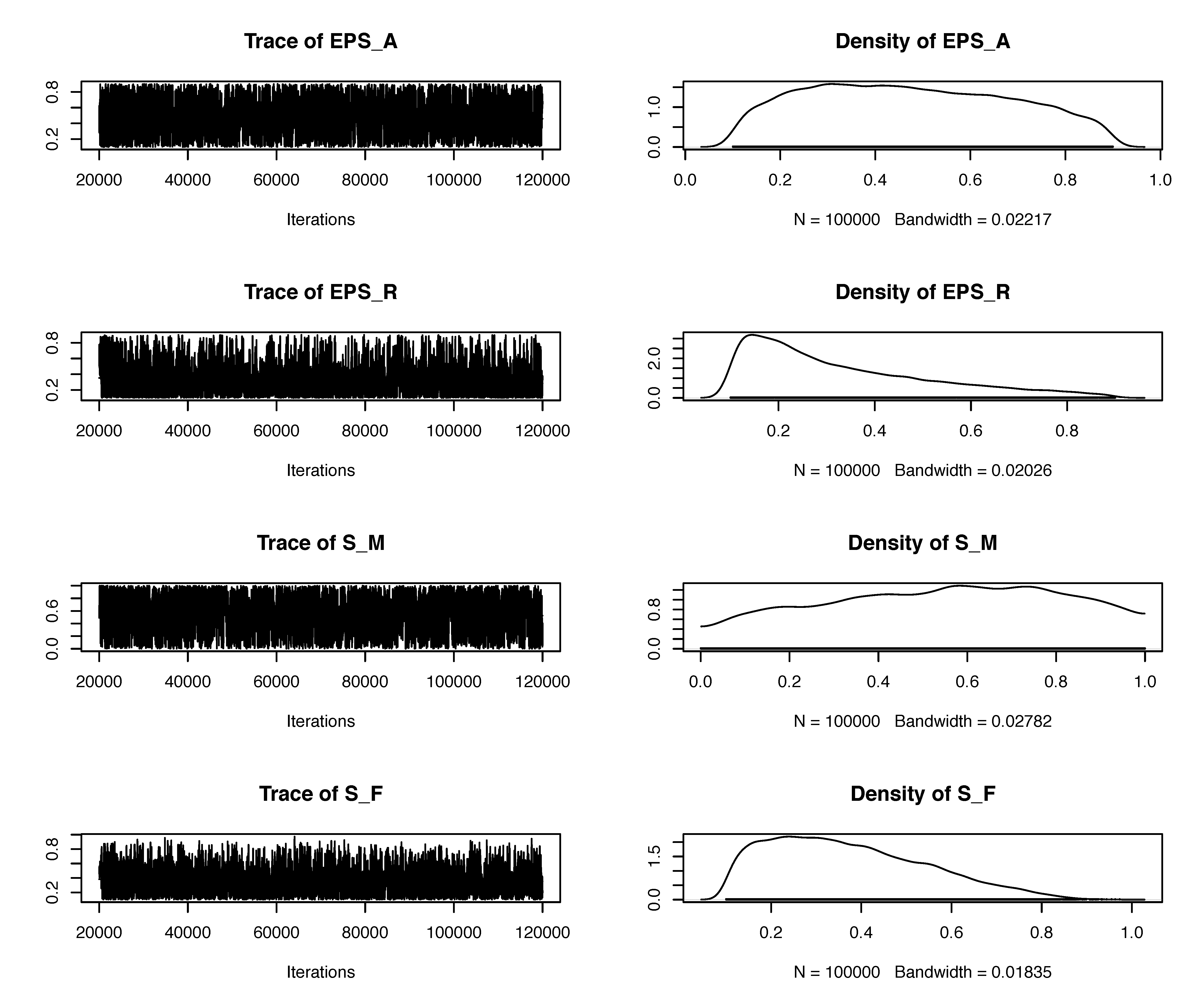
**

| **Parameter** | **Prior distribution (chosen based on literature)** | **Posterior distribution** | | | |
| --- | --- | --- | --- | --- | --- |
|  |  | **mean** | **SD** | **median** | **HPD interval (95%)** |
| 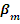 | **U(0.10-1.00)** | **0.499** | **0.205** | **0.476** | **0.155 - 0.889** |
| 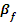 | **U(0.10,1.00)** | **0.726** | **0.162** | **0.740** | **0.438 – 0.999** |
| 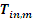 | **U(0.60,1.70)** | **1.268** | **0.270** | **1.298** | **0.783 – 1.700** |
| 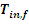 | **U(0.75,1.50)** | **1.291** | **0.151** | **1.324** | **0.986 – 1.500** |
| 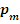 | **U(0.01,0.30)** | **0.140** | **0.057** | **0.129** | **0.103 – 0.827** |
| 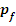 | **U(0.40,0.70)** | **0.477** | **0.062** | **0.462** | **0.400 – 0.604** |
| 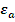 | **U(0.10,0.90)** | **0.474** | **0.209** | **0.462** | **0.103 – 0.827** |
| 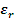 | **U(0.10,0.90)** | **0.331** | **0.191** | **0.275** | **0.100 – 0.727** |
| 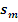 | **U(0.00,1.00)** | **0.533** | **0.262** | **0.553** | **0.068 – 0.974** |
| 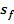 | **U(0.10,1.00)** | **0.371** | **0.173** | **0.348** | **0.100 – 0.694** |

**Model SIS_2_**


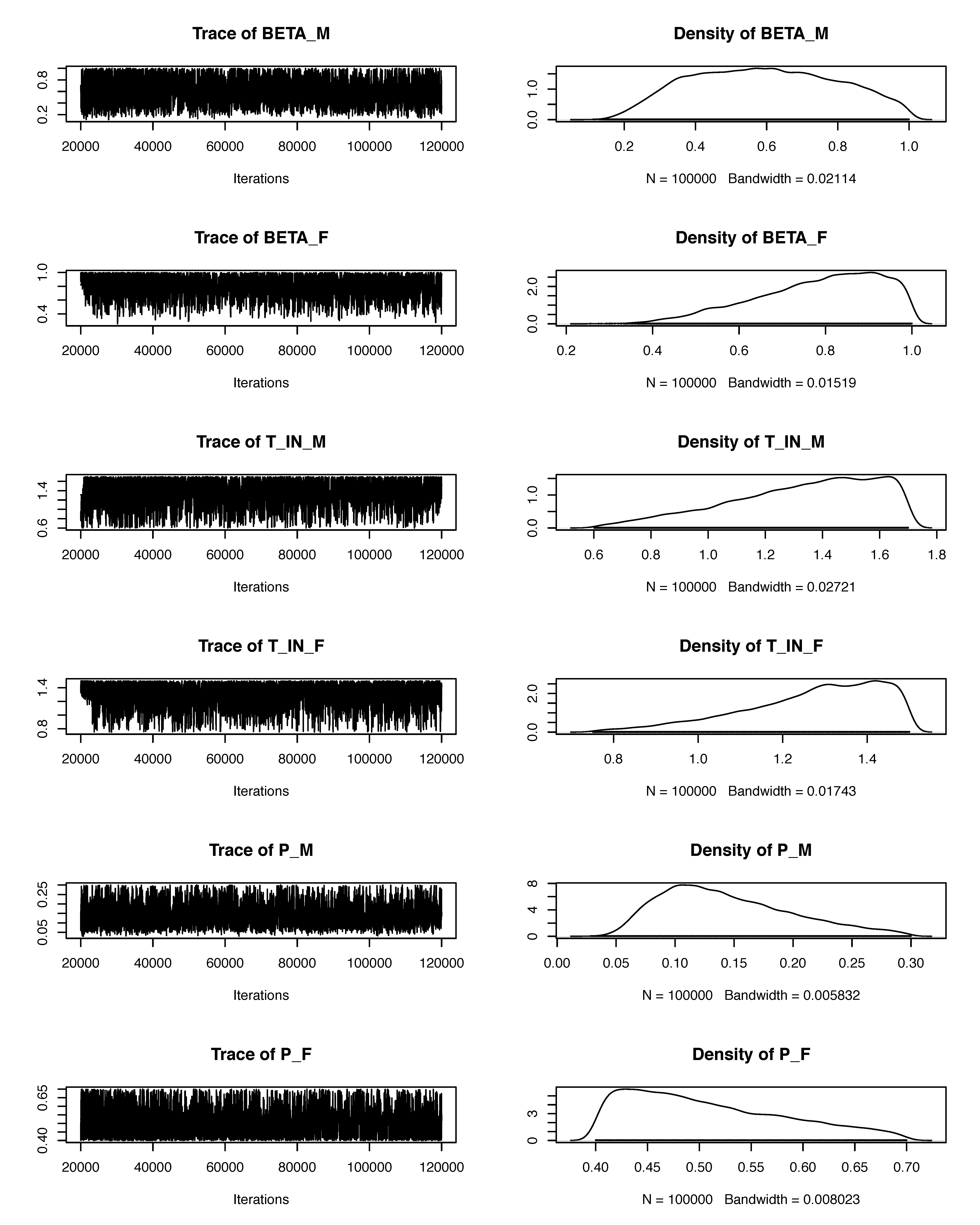


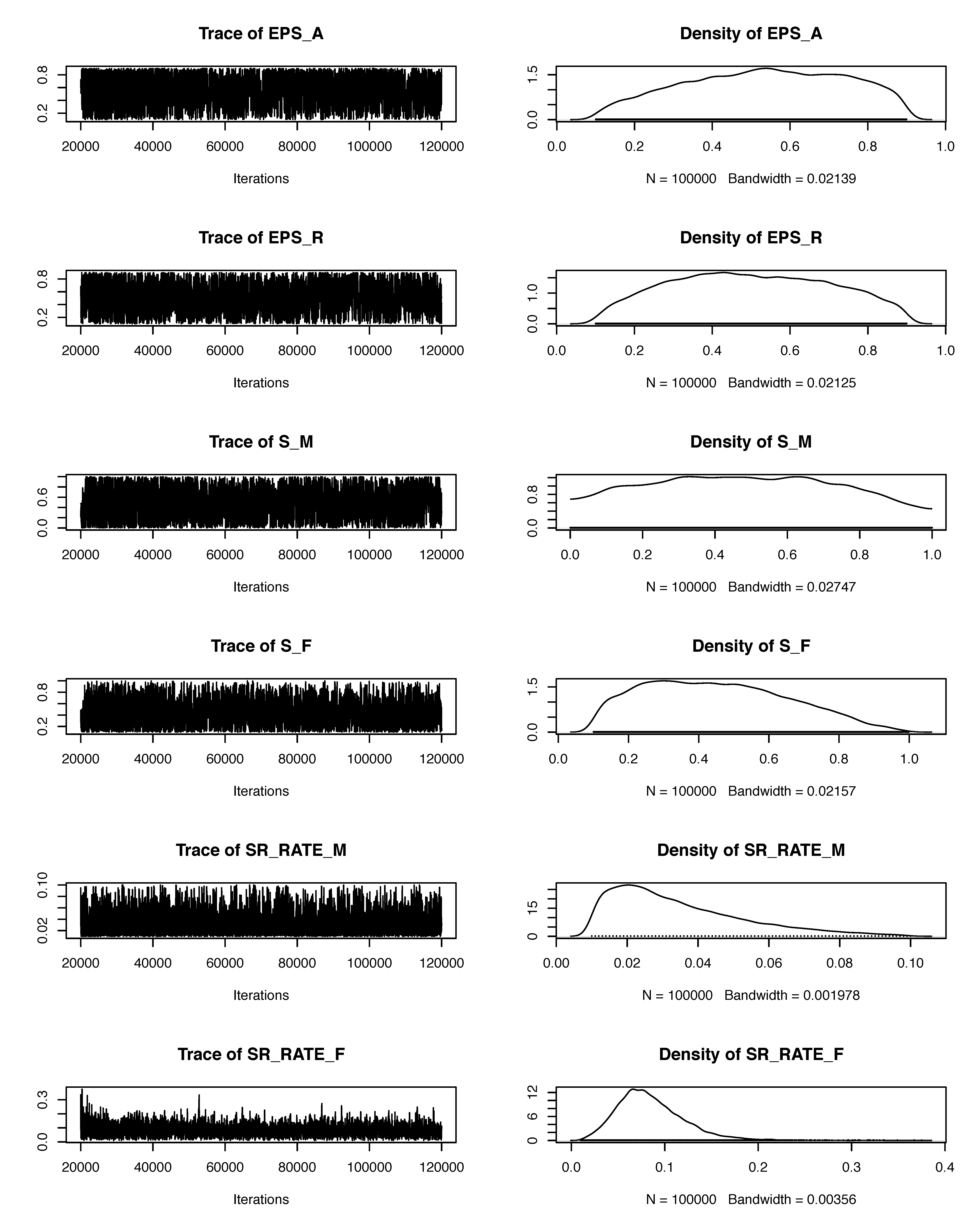


| **Parameter** | **Prior distribution (chosen based on literature)** | **Posterior distribution** | | | |
| --- | --- | --- | --- | --- | --- |
|  |  | **mean** | **SD** | **median** | **HPD interval (95%)** |
| 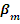 | **U(0.10-1.00)** | **0.594** | **0.199** | **0.590** | **0.248 – 0.961** |
| 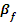 | **U(0.10,1.00)** | **0.782** | **0.143** | **0.806** | **0.514 – 0.999** |
| 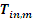 | **U(0.60,1.70)** | **1.324** | **0.256** | **1.367** | **0.837 – 1.700** |
| 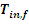 | **U(0.75,1.50)** | **1.268** | **0.164** | **1.300** | **0.945 – 1.500** |
| 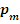 | **U(0.01,0.30)** | **0.144** | **0.055** | **0.135** | **0.056 – 0.257** |
| 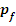 | **U(0.40,0.70)** | **0.508** | **0.075** | **0.494** | **0.400 – 0.654** |
| 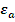 | **U(0.10,0.90)** | **0.534** | **0.201** | **0.541** | **0.183 – 0.891** |
| 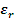 | **U(0.10,0.90)** | **0.500** | **0.200** | **0.495** | **0.143 – 0.856** |
| 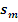 | **U(0.00,1.00)** | **0.480** | **0.259** | **0.477** | **0.001 – 0.903** |
| 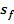 | **U(0.10,1.00)** | **0.452** | **0.203** | **0.438** | **0.100 – 0.810** |
| 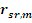 | **U(0.01,0.10)** | **0.034** | **0.018** | **0.030** | **0.010 – 0.073** |
| 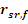 | **U(0.01,0.50)** | **0.084** | **0.036** | **0.080** | **0.019 – 0.153** |

**Model SIR_1_**


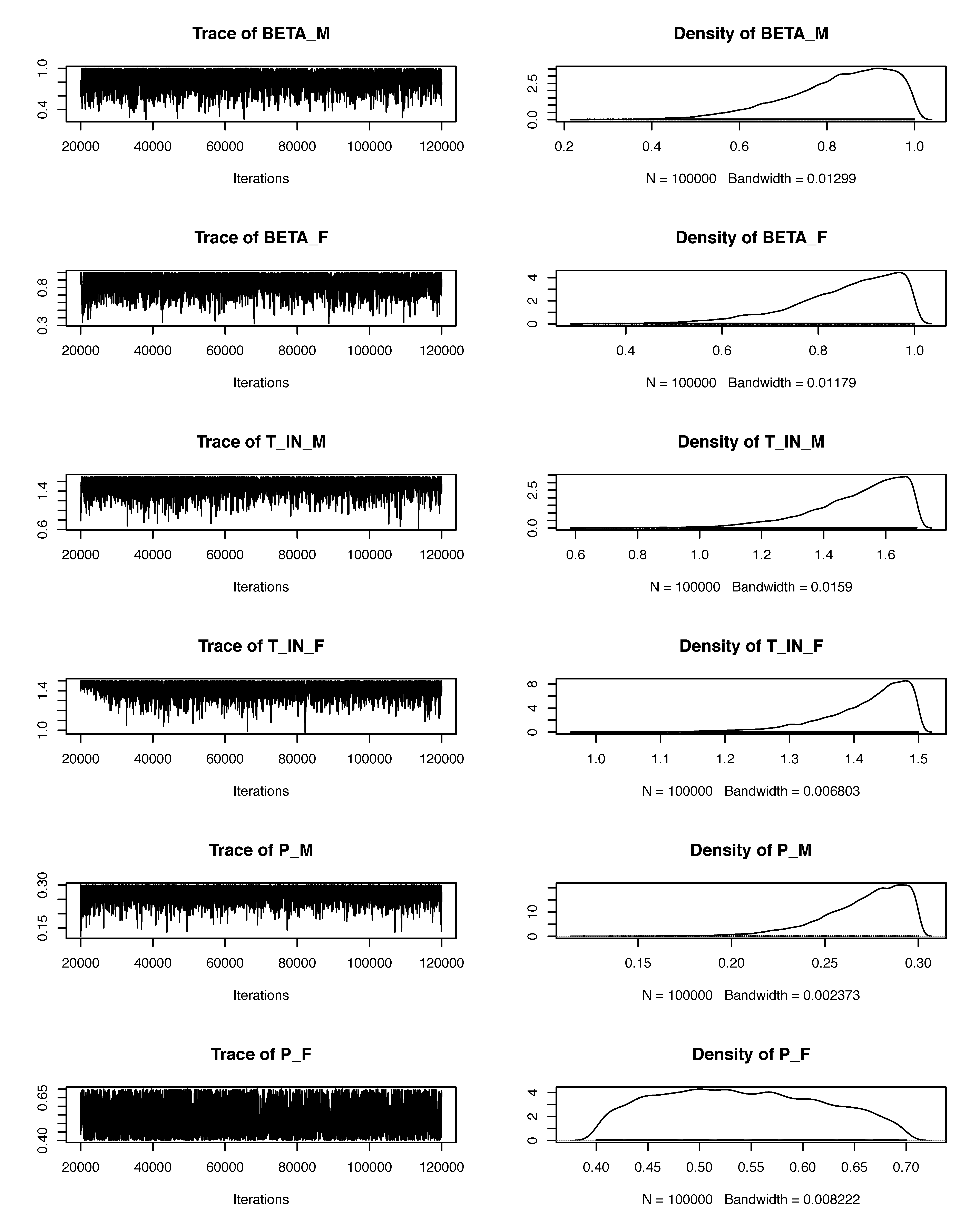


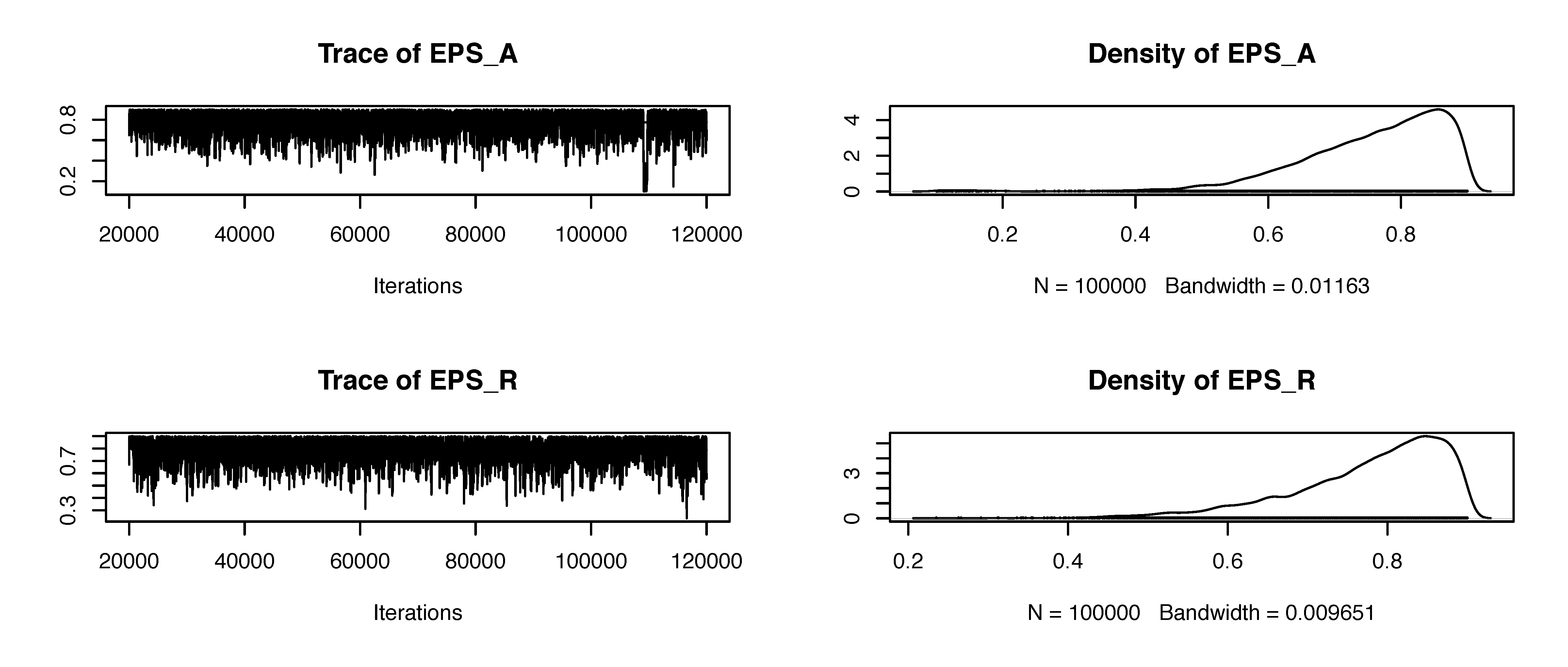


| **Parameter** | **Prior distribution (chosen based on literature)** | **Posterior distribution** | | | |
| --- | --- | --- | --- | --- | --- |
|  |  | **mean** | **SD** | **median** | **HPD interval (95%)** |
| 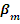 | **U(0.10-1.00)** | **0.825** | **0.122** | **0.847** | **0.589 – 0.999** |
| 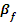 | **U(0.10,1.00)** | **0.855** | **0.112** | **0.880** | **0.633 – 1.000** |
|  | **U(0.60,1.70)** | **1.506** | **0.157** | **1.544** | **1.195 – 1.700** |
|  | **U(0.75,1.50)** | **1.418** | **0.070** | **1.438** | **1.278 – 1.500** |
|  | **U(0.01,0.30)** | **0.270** | **0.023** | **0.276** | **0.225 – 0.300** |
|  | **U(0.40,0.70)** | **0.540** | **0.077** | **0.536** | **0.401 – 0.671** |
|  | **U(0.10,0.90)** | **0.756** | **0.112** | **0.780** | **0.559 – 0.900** |
|  | **U(0.10,0.90)** | **0.777** | **0.094** | **0.800** | **0.589 – 0.900** |

**Model SIR_2_**

| **Parameter** | **Prior distribution (chosen based on literature)** | **Posterior distribution** | | | |
| --- | --- | --- | --- | --- | --- |
|  |  | **mean** | **SD** | **median** | **HPD interval (95%)** |
|  | **U(0.10-1.00)** | **0.769** | **0.140** | **0.786** | **0.515 – 0.999** |
|  | **U(0.10,1.00)** | **0.862** | **0.106** | **0.885** | **0.654 – 1.000** |
|  | **U(0.60,1.70)** | **1.510** | **0.160** | **1.551** | **1.190 – 1.700** |
|  | **U(0.75,1.50)** | **1.413** | **0.074** | **1.433** | **1.269 – 1.500** |
|  | **U(0.01,0.30)** | **0.254** | **0.032** | **0.261** | **0.191 – 0.300** |
|  | **U(0.40,0.70)** | **0.527** | **0.076** | **0.520** | **0.401 – 0.665** |
|  | **U(0.10,0.90)** | **0.782** | **0.091** | **0.803** | **0.605 – 0.900** |
|  | **U(0.10,0.90)** | **0.762** | **0.104** | **0.787** | **0.552 – 0.900** |
|  | **U(0.50,0.95)** | **0.683** | **0.113** | **0.669** | **0.501 – 0.892** |
|  | **U(0.50,0.95)** | **0.728** | **0.119** | **0.729** | **0.523 – 0.935** |

**Model SIRS_1_**

| **Parameter** | **Prior distribution (chosen based on literature)** | **Posterior distribution** | | | |
| --- | --- | --- | --- | --- | --- |
|  |  | **mean** | **SD** | **median** | **HPD interval (95%)** |
|  | **U(0.10-1.00)** | **0.842** | **0.117** | **0.867** | **0.614 – 1.000** |
|  | **U(0.10,1.00)** | **0.917** | **0.068** | **0.934** | **0.780 – 1.000** |
|  | **U(0.60,1.70)** | **1.557** | **0.121** | **1.588** | **1.316 – 1.700** |
|  | **U(0.75,1.50)** | **1.398** | **0.084** | **1.420** | **1.235 – 1.500** |
|  | **U(0.01,0.30)** | **0.267** | **0.024** | **0.273** | **0.218 – 0.300** |
|  | **U(0.40,0.70)** | **0.597** | **0.069** | **0.610** | **0.463 – 0.700** |
|  | **U(0.10,0.90)** | **0.659** | **0.154** | **0.681** | **0.367 – 0.899** |
|  | **U(0.10,0.90)** | **0.765** | **0.107** | **0.792** | **0.550 – 0.900** |
|  | **U(0.01,0.33)** | **0.056** | **0.044** | **0.044** | **0.010 – 0.150** |
|  | **U(0.01,0.33)** | **0.129** | **0.051** | **0.121** | **0.041 – 0.238** |

**Model SIRS_2_**

| **Parameter** | **Prior distribution (chosen based on literature)** | **Posterior distribution** | | | |
| --- | --- | --- | --- | --- | --- |
|  |  | **mean** | **SD** | **median** | **HPD interval (95%)** |
|  | **U(0.10-1.00)** | **0.828** | **0.126** | **0.855** | **0.578 – 1.000** |
|  | **U(0.10,1.00)** | **0.915** | **0.068** | **0.932** | **0.779 – 1.000** |
|  | **U(0.60,1.70)** | **1.553** | **0.126** | **1.586** | **1.296 – 1.700** |
|  | **U(0.75,1.50)** | **1.395** | **0.086** | **1.418** | **1.220 – 1.500** |
|  | **U(0.01,0.30)** | **0.254** | **0.033** | **0.261** | **0.190 – 0.300** |
|  | **U(0.40,0.70)** | **0.583** | **0.073** | **0.594** | **0.448 – 0.699** |
|  | **U(0.10,0.90)** | **0.682** | **0.151** | **0.708** | **0.390 – 0.900** |
|  | **U(0.10,0.90)** | **0.748** | **0.113** | **0.774** | **0.524 – 0.900** |
|  | **U(0.50,0.95)** | **0.654** | **0.108** | **0.633** | **0.500 – 0.865** |
|  | **U(0.50,0.95)** | **0.674** | **0.116** | **0.653** | **0.500 – 0.893** |
|  | **U(0.01,0.33)** | **0.116** | **0.083** | **0.091** | **0.010 – 0.285** |
|  | **U(0.01,0.33)** | **0.162** | **0.061** | **0.154** | **0.054 – 0.285** |

**Model SIRS_3_**

| **Parameter** | **Prior distribution (chosen based on literature)** | **Posterior distribution** | | | |
| --- | --- | --- | --- | --- | --- |
|  |  | **mean** | **SD** | **median** | **HPD interval (95%)** |
|  | **U(0.10-1.00)** | **0.593** | **0.207** | **0.586** | **0.246 – 0.968** |
|  | **U(0.10,1.00)** | **0.794** | **0.137** | **0.817** | **0.535 – 1.000** |
|  | **U(0.60,1.70)** | **1.346** | **0.243** | **1.392** | **0.883 – 1.700** |
|  | **U(0.75,1.50)** | **1.339** | **0.123** | **1.367** | **1.097 – 1.500** |
|  | **U(0.01,0.30)** | **0.166** | **0.053** | **0.159** | **0.075 – 0.273** |
|  | **U(0.40,0.70)** | **0.487** | **0.066** | **0.471** | **0.400 – 0.624** |
|  | **U(0.10,0.90)** | **0.453** | **0.207** | **0.438** | **0.100 – 0.813** |
|  | **U(0.10,0.90)** | **0.502** | **0.200** | **0.502** | **0.158 – 0.869** |
|  | **U(0.01,1.00)** | **0.651** | **0.215** | **0.675** | **0.259 – 0.999** |
|  | **U(0.01,1.00)** | **0.693** | **0.195** | **0.719** | **0.332 – 0.999** |

**Model SIRS_4_**

| **Parameter** | **Prior distribution (chosen based on literature)** | **Posterior distribution** | | | |
| --- | --- | --- | --- | --- | --- |
|  |  | **mean** | **SD** | **median** | **HPD interval (95%)** |
|  | **U(0.10-1.00)** | **0.679** | **0.182** | **0.695** | **0.362 – 0.999** |
|  | **U(0.10,1.00)** | **0.862** | **0.102** | **0.885** | **0.660 – 0.999** |
|  | **U(0.60,1.70)** | **1.436** | **0.197** | **1.480** | **1.046 – 1.700** |
|  | **U(0.75,1.50)** | **1.340** | **0.122** | **1.367** | **1.098 – 1.500** |
|  | **U(0.01,0.30)** | **0.172** | **0.052** | **0.167** | **0.085 – 0.276** |
|  | **U(0.40,0.70)** | **0.523** | **0.078** | **0.513** | **0.400 – 0.664** |
|  | **U(0.10,0.90)** | **0.478** | **0.204** | **0.473** | **0.100 – 0.823** |
|  | **U(0.10,0.90)** | **0.656** | **0.160** | **0.681** | **0.354 – 0.900** |
|  | **U(0.01,1.00)** | **0.716** | **0.185** | **0.745** | **0.365 – 0.999** |
|  | **U(0.01,1.00)** | **0.732** | **0.176** | **0.762** | **0.403 – 1.000** |
|  | **U(0.01,0.10)** | **0.033** | **0.017** | **0.030** | **0.010 – 0.069** |
|  | **U(0.01,0.50)** | **0.083** | **0.032** | **0.079** | **0.022 – 0.147** |

**Technical Appendix**

**Igor A. Korostil^1^, Suzanne M. Garland^2, 3, 4^, Matthew G. Law^1^ and David G. Regan^1^**

^1^The Kirby Institute, University of New South Wales, Sydney, NSW 2052, Australia

^2^Regional World Health Organization Human Papillomavirus Laboratory Network, Department of Microbiology and Infectious Diseases, The Royal Women’s Hospital, Melbourne, VIC 3052, Australia

^3^Department of Obstetrics and Gynaecology, University of Melbourne, VIC 3052, Australia

^4^Murdoch Childrens Research Institute, VIC 3052, Australia

## Model equations

All equations use brackets to denote the number of individuals in a state. Each state is stratified by gender, sexual activity group and age group (not shown explicitly to simplify notations). Equations are solved sequentially on time intervals [0,1] which correspond to 1 year. An initial condition imposed at the beginning of each time interval is that the number of individuals of age in each state is the number of individuals aged calculated at the end of the previous interval (this way we implement the process of aging). The exception to this is the first age group, to which we simply add new susceptible individuals at the beginning of each interval.

### Model SIS_1_

### Model SIS_2_

### Model SIR_1_

### Model SIR_2_

### Model SIRS_1_

### Model SIRS_2_

### Model SIRS_3_

### Model SIRS_4_

## Calculation of the force of infection

Force of infection, usually denoted , is a rate at which an individual becomes infected per unit time. In our models it is a yearly rate. For example, the force of infection on females of a given age and sexual activity level is defined as

where is a probability of HPV transmission from male to female per partnership, is a mean partner change rate for females of this category, is a probability that these females get male partners of a particular age and sexual activity who are infected with probability (which is assumed to be equal to HPV prevalence in males of the category in question). Note that HVP prevalence is the proportion of all (both seropositive and seronegative) individuals testing DNA positive.

In this paper we use the implementation of sexual mixing developed by Garnett and Anderson . The sexual behavior data required for this implementation (originally presented in and derived based on the ASHR survey results ) are as below.

| Sexual activity group | 1 | 2 | 3 | 4 |
| --- | --- | --- | --- | --- |
| Percent of population in the group | 60 | 27 | 11 | 2 |
| Relative yearly partner change rate | 1 | 4.76 | 24.83 | 105.65 |

| Age group | 16-19 | 20-24 | 25-29 | 30-34 | 35-39 | 40-44 | 45-59 |
| --- | --- | --- | --- | --- | --- | --- | --- |
| Sexual partner change rate, per year | 5.28 | 6.06 | 4.37 | 2.57 | 1.61 | 1.43 | 1 |

## The overall annual sexual partner change rate for the entire Australian sexually active population was fixed at 0.43.

## Selection of prior distributions

**Per-partnership probability of HPV-16 transmission from female to male,**  and from male to female,

An estimation of this probability at 0.20 (95% CI, 0.16–0.24) was reported by the HITCH Study (HPV Infection and Transmission among Couples through Heterosexual activity) . The study enrolled 18-24 y.o. women and their partners, 179 discordant couples in total. No notable differences were detected between the probabilities female-to-male and male-to-female transmissions.

However, taking into account a considerable uncertainty surrounding this probability, especially, in the context of compartmental models where all partnerships are treated as instantaneous, we decided to use the reported estimation to define only the lower boundary of the prior, that is, we have assigned a uniform distribution U(0.1,1.0) to both and .

**Average infection for females,**

We selected as our primary source the extensive Ludwig-McGill cohort study which recruited 2,462 women from Brazil aged 18-60. The study reported mean duration of 11.9 (10.3–13.5) (or in years 0.99 (0.86-1.12)) and median 7.3 (6.3–10.7) (in years 0.6 (0.52-0.9)).

A cohort of 331 women aged 18-35 years was examined within the Young Women’s Health Study in the USA . The median duration of infection was found to be 9.8 months (0.81 years).

On the other hand, a Canadian study which recruited 635 female university students attending either the McGill or the Concordia University Health Clinic reported notably longer mean duration of infection of 18.3 months (95% CI: 12.9-23.7), or 1.52 (1.07-1.97) in years.

Given this information, we selected a uniform prior distribution U(0.75,1.5) for this parameter.

**Average duration of infection for males,**

A study conducted in Tucson, USA covered 290 men aged 18 – 44 years and reported a median duration of 6 months (95% CI: 5.2-6.8), which is in years 0.5 (0.43-0.57) . The HPV in Men (HIM) study (1159 men aged 18-70 years from USA, Brazil and Mexico) derived the median duration of 12.19 months (95% CI: 7.16-18.17) or 1.01 (0.6-1.56) in years .

To select a prior for the mean duration of infection we assumed that just like for females, the mean is likely to be greater than median, and selected U(0.6,1.7) as a prior.

**Average rate of loss of natural immunity for males,**  and **females,**

We distinguish between two cases here. One is when seropositivity indicates full immunity (models SIRS_1_  and SIRS_2_ ). In these models an individual who is seropositive can not become re-infected, so the loss of immunity is simultaneous with the loss of seropositivity. This implies that though we have no data on the duration of immunity, we do have some on the duration of seropositivity which we can use. In particular, the reported minimal durations of seropositivity vary from 3 to 7-13 years , which lets us assume the minimal duration of natural immunity to be 3 years. For males, we assumed the same minimal durations by analogy.

The second case is when seropositivity is not limited to the immune state: an individual can become susceptible or infected while still remaining seropositive (models SIRS_3_  and SIRS_4_ ). Then there is no reason to restrict the minimal duration of immunity to at least 3 years, so we assumed it is equally likely to be from 1 year to 100 years.

In our models the actual parameter we used was the rate of loss of immunity, defined as 1/. Given the considerations above, this rate was uniformly distributed according to U(0.01,0.33) for case one, and U(0.01,1.00) for case two.

**Probability of seroconversion for males,**

A recent study covering 18-21 y.o. male students (156 in total) recruited at the University of Washington in Seattle, at 2 years from first detection of genital HPV infection percentage of seroconverted was estimated at 13.0 (95% CI: 6.6-24.8).

Based on this, we selected a uniform prior U(0.01,0.3).

**Probability of seroconversion for females,**

Females university students aged 18-20 were studied in . About 50% of them seroconverted at 1 year after the first HPV-16 DNA detection, and at 2 years the percentage increased to about 60%,. Then it stayed at approximately the same level.

In view of this, we selected a uniform prior distribution U(0.4,0.7) for this parameter.

**Rate of seroreversion for males,**

A study covering 809 females and 768 males over 14 was performed within the project RESPECT . At baseline, 147 out of 768 males were seropositive, and at 1 year of follow up, the number of seropositive males decreased to 139, which is a 5.44% reduction.

Hence, we selected a wide uniform prior U(0.01,0.1), i.e. 1% to 10%.

**Rate of seroreversion for females,**

The Finnish Family HPV Study reported antibody decay in 290 women (mean age 25.5 years). During the median follow-up time (37.2 months), decay of antibodies to HPV 16 was observed in 5.3% of women.

Females university students aged 18-20 residing in the state of Washington were studied in . It was observed that 20 (71.4%) of 28 women HPV-16 seropositive at all visits (the average length of follow-up was 31.3 months).

A study conducted in Guanacaste, Costa Rica reported that 55% (675 of 1216) of women seropositive at enrolment (1993-94) remained seropositive for HPV-16 at follow-up (2000).

To ensure that these somewhat conflicting data would be taken into account in our comparison, we chose a uniform prior distribution U(0.01,0.5).

**Degree of natural immunity for seropositive males,**

Since no data were available to inform the choice of a prior distributions for this parameter, we selected U(0.0,1.0), that is, the degree was assumed to be equally likely anywhere in the range from 0 (non-existent) to 1 (full immunity).

**Degree of natural immunity for seropositive females,**

A total of 151 incident HPV16 infections were observed in a cohort of 974 women from the Guanacaste Natural History Study . Both VLP ELISA and cLIA were associated with protection against subsequent HPV infections. Although the point estimate of the cLIA result showed stronger protection, the difference was not significant (HPV16 cLIA: OR, 0.44 [95% CI, 0.21–0.93]; HPV16 VLP ELISA: OR, 0.56 [95% CI, 0.33–0.93]; sampling-adjusted estimate for HPV16 cLIA: OR, 0.37 [95% CI, 0.15–0.94]; sampling- adjusted estimate for HPV16 VLP ELISA: OR, 0.54 [95% CI, 0.29–1.03]).

A study based at Rutgers University recruited 608 female students (mean age 20) and concluded that for the subjects who had persistently high levels of IgG to HPV-16 VLPs for >2 visits, their relative risk for subsequent infection was close to zero.

A Costa Rican study with women aged 18–25 years at enrollment observed that having high HPV16 antibody titer at enrollment was associated with a reduced risk of subsequent HPV16 infection (women in the highest tertile of HPV16 antibody titers, adjusted rate ratio 0.50, 95% CI 0.26-0.86 vs HPV16-seronegative women).

This information can be interpreted as an indication that there is a nonzero degree of immunity for females, which is likely to be significant. To make sure that the reasonably widest range of possible values is covered, we chose a uniform distribution U(0.1,1.0).

**Average time to seroconversion for females,**

A study covering 809 females and 768 males over 14 was performed within the project RESPECT . Of 229 cases of incident HPV-16 seropositivity, 137 (59.8%) had seroconverted by the 6-month follow-up visit and 92 (40.2%) had seroconverted by the 12-month follow-up visit.

Females university students aged 18-20 residing in the state of Washington were studied in . For 42 subjects under observation, the median time to seroconversion from DNA detection was 11.8 months (0.98 years).

This parameter was actually implemented as a fraction of the average duration of infection, which appears to be at least half of it. Hence we used a uniform distribution U(0.5,0.95).

**Average time to seroconversion for males,**

Based on the results of a recent study covering 18-21 y.o. male students (156 in total), we, just like for females, implemented this time as a fraction of the average duration of infection drawn from a uniform distribution U(0.5,0.95).

**Average time to clear infection following seroconversion for females or males,**

This time is not a separate parameter in the models (SIR_2_ and SIRS_2_), it is only used as a notation. In fact, it is equal to the average duration of infection minus the average time to seroconversion.

**Sexual mixing parameters**

All our models had two sexual mixing parameters: degrees of assortativity by age () and sexual activity group (). These were assigned the following prior uniform distributions: ~U(0.1,0.9) and ~U(0.1,0.9).

## Data used to calibrate the models

**Seroprevalence data**

We used the age-specific seroprevalence data reported in . While the detailed discussion on the data and methods of its collection are provided in the paper we refer to, here we briefly mention some of that information.

To collect serum samples, public and private laboratories from New South Wales, Victoria, and Queensland were contacted. These 3 states account for about 80% of Australian population. Recorded demographic data were the following: age group, sex, and date of sample collection. The age groups were specified as 0–4, 5–9, 10–14, 15–19, 20–29, 30–39, 40–49, 50–59, and 60–69 years of age.

The sample size was calculated taking into account the mean cumulative number of lifetime sexual partners by age cohort, as reported by the Australian Study of Health and Relationships which we use in our study. The number of samples from females was 1,523 and from males 1,247.

The samples were tested at Merck Research Laboratories (Wayne, Pennsylvania). The overall population HPV seroprevalence was estimated via weighting to Australian population estimates by age.

## HPV DNA prevalence data for females

The seroprevalence data we employed were from the recent the Women’s HPV Indigenous Non-Indigenous Urban Rural Study (WHINURS) study as reported in . The study covered 655 Indigenous and 1,494 non-Indigenous women aged 18 to 40 years who were attending their usual healthcare provider for routine Pap smear cytology . Women represented all Australian states and one territory (Northern Territory) of Australia and the data were obtained from 16 Indigenous health services, 8 family-planning services and 10 community clinics.

Age-adjusted prevalences were calculated by weighting the WHINURS sample to the relevant Australian Bureau of Statistics (ABS) population structure, by single year of age.

Among the key limitations, apart from not inclusion of women over 40, are that the sample was not geographically or demographically representative of all Australian women and non-Indigenous women who attended free community health services, were often likely to be of lower socioeconomic status on average than other Australian women (see for discussion).

**MCMC trace plots, posterior density plots, quantitative summaries of posterior distributions and calibration plots for all models**

We obtained 120,000 samples from posterior distributions for each model parameter. MCMC trace plots, posterior density plots, posterior means, medians, etc. were produced using the R package CODA (Output analysis and diagnostics for Markov Chain Monte Carlo simulations).

Calibration plots show the actual data (means) with 95% confidence intervals (gray whiskers) as well as the simulated means with 0.975 and 0.025 quantiles over the last 100,000 of MCMC samples.

Finally, the 95% Highest Posterior Density (HPD) interval presented here is the shortest interval in parameter space which contains 95% of the distribution.

**Model SIS_1_**

| **Parameter** | **Prior distribution (chosen based on literature)** | **Posterior distribution** | | | |
| --- | --- | --- | --- | --- | --- |
|  |  | **mean** | **SD** | **median** | **HPD interval (95%)** |
|  | **U(0.10-1.00)** | **0.499** | **0.205** | **0.476** | **0.155 - 0.889** |
|  | **U(0.10,1.00)** | **0.726** | **0.162** | **0.740** | **0.438 – 0.999** |
|  | **U(0.60,1.70)** | **1.268** | **0.270** | **1.298** | **0.783 – 1.700** |
|  | **U(0.75,1.50)** | **1.291** | **0.151** | **1.324** | **0.986 – 1.500** |
|  | **U(0.01,0.30)** | **0.140** | **0.057** | **0.129** | **0.103 – 0.827** |
|  | **U(0.40,0.70)** | **0.477** | **0.062** | **0.462** | **0.400 – 0.604** |
|  | **U(0.10,0.90)** | **0.474** | **0.209** | **0.462** | **0.103 – 0.827** |
|  | **U(0.10,0.90)** | **0.331** | **0.191** | **0.275** | **0.100 – 0.727** |
|  | **U(0.00,1.00)** | **0.533** | **0.262** | **0.553** | **0.068 – 0.974** |
|  | **U(0.10,1.00)** | **0.371** | **0.173** | **0.348** | **0.100 – 0.694** |

**Model SIS_2_**

| **Parameter** | **Prior distribution (chosen based on literature)** | **Posterior distribution** | | | |
| --- | --- | --- | --- | --- | --- |
|  |  | **mean** | **SD** | **median** | **HPD interval (95%)** |
|  | **U(0.10-1.00)** | **0.594** | **0.199** | **0.590** | **0.248 – 0.961** |
|  | **U(0.10,1.00)** | **0.782** | **0.143** | **0.806** | **0.514 – 0.999** |
|  | **U(0.60,1.70)** | **1.324** | **0.256** | **1.367** | **0.837 – 1.700** |
|  | **U(0.75,1.50)** | **1.268** | **0.164** | **1.300** | **0.945 – 1.500** |
|  | **U(0.01,0.30)** | **0.144** | **0.055** | **0.135** | **0.056 – 0.257** |
|  | **U(0.40,0.70)** | **0.508** | **0.075** | **0.494** | **0.400 – 0.654** |
|  | **U(0.10,0.90)** | **0.534** | **0.201** | **0.541** | **0.183 – 0.891** |
|  | **U(0.10,0.90)** | **0.500** | **0.200** | **0.495** | **0.143 – 0.856** |
|  | **U(0.00,1.00)** | **0.480** | **0.259** | **0.477** | **0.001 – 0.903** |
|  | **U(0.10,1.00)** | **0.452** | **0.203** | **0.438** | **0.100 – 0.810** |
|  | **U(0.01,0.10)** | **0.034** | **0.018** | **0.030** | **0.010 – 0.073** |
|  | **U(0.01,0.50)** | **0.084** | **0.036** | **0.080** | **0.019 – 0.153** |

**Model SIR_1_**

| **Parameter** | **Prior distribution (chosen based on literature)** | **Posterior distribution** | | | |
| --- | --- | --- | --- | --- | --- |
|  |  | **mean** | **SD** | **median** | **HPD interval (95%)** |
|  | **U(0.10-1.00)** | **0.825** | **0.122** | **0.847** | **0.589 – 0.999** |
|  | **U(0.10,1.00)** | **0.855** | **0.112** | **0.880** | **0.633 – 1.000** |
|  | **U(0.60,1.70)** | **1.506** | **0.157** | **1.544** | **1.195 – 1.700** |
|  | **U(0.75,1.50)** | **1.418** | **0.070** | **1.438** | **1.278 – 1.500** |
|  | **U(0.01,0.30)** | **0.270** | **0.023** | **0.276** | **0.225 – 0.300** |
|  | **U(0.40,0.70)** | **0.540** | **0.077** | **0.536** | **0.401 – 0.671** |
|  | **U(0.10,0.90)** | **0.756** | **0.112** | **0.780** | **0.559 – 0.900** |
|  | **U(0.10,0.90)** | **0.777** | **0.094** | **0.800** | **0.589 – 0.900** |

**Model SIR_2_**

| **Parameter** | **Prior distribution (chosen based on literature)** | **Posterior distribution** | | | |
| --- | --- | --- | --- | --- | --- |
|  |  | **mean** | **SD** | **median** | **HPD interval (95%)** |
|  | **U(0.10-1.00)** | **0.769** | **0.140** | **0.786** | **0.515 – 0.999** |
|  | **U(0.10,1.00)** | **0.862** | **0.106** | **0.885** | **0.654 – 1.000** |
|  | **U(0.60,1.70)** | **1.510** | **0.160** | **1.551** | **1.190 – 1.700** |
|  | **U(0.75,1.50)** | **1.413** | **0.074** | **1.433** | **1.269 – 1.500** |
|  | **U(0.01,0.30)** | **0.254** | **0.032** | **0.261** | **0.191 – 0.300** |
|  | **U(0.40,0.70)** | **0.527** | **0.076** | **0.520** | **0.401 – 0.665** |
|  | **U(0.10,0.90)** | **0.782** | **0.091** | **0.803** | **0.605 – 0.900** |
|  | **U(0.10,0.90)** | **0.762** | **0.104** | **0.787** | **0.552 – 0.900** |
|  | **U(0.50,0.95)** | **0.683** | **0.113** | **0.669** | **0.501 – 0.892** |
|  | **U(0.50,0.95)** | **0.728** | **0.119** | **0.729** | **0.523 – 0.935** |

**Model SIRS_1_**

| **Parameter** | **Prior distribution (chosen based on literature)** | **Posterior distribution** | | | |
| --- | --- | --- | --- | --- | --- |
|  |  | **mean** | **SD** | **median** | **HPD interval (95%)** |
|  | **U(0.10-1.00)** | **0.842** | **0.117** | **0.867** | **0.614 – 1.000** |
|  | **U(0.10,1.00)** | **0.917** | **0.068** | **0.934** | **0.780 – 1.000** |
|  | **U(0.60,1.70)** | **1.557** | **0.121** | **1.588** | **1.316 – 1.700** |
|  | **U(0.75,1.50)** | **1.398** | **0.084** | **1.420** | **1.235 – 1.500** |
|  | **U(0.01,0.30)** | **0.267** | **0.024** | **0.273** | **0.218 – 0.300** |
|  | **U(0.40,0.70)** | **0.597** | **0.069** | **0.610** | **0.463 – 0.700** |
|  | **U(0.10,0.90)** | **0.659** | **0.154** | **0.681** | **0.367 – 0.899** |
|  | **U(0.10,0.90)** | **0.765** | **0.107** | **0.792** | **0.550 – 0.900** |
|  | **U(0.01,0.33)** | **0.056** | **0.044** | **0.044** | **0.010 – 0.150** |
|  | **U(0.01,0.33)** | **0.129** | **0.051** | **0.121** | **0.041 – 0.238** |

**Model SIRS_2_**

| **Parameter** | **Prior distribution (chosen based on literature)** | **Posterior distribution** | | | |
| --- | --- | --- | --- | --- | --- |
|  |  | **mean** | **SD** | **median** | **HPD interval (95%)** |
|  | **U(0.10-1.00)** | **0.828** | **0.126** | **0.855** | **0.578 – 1.000** |
|  | **U(0.10,1.00)** | **0.915** | **0.068** | **0.932** | **0.779 – 1.000** |
|  | **U(0.60,1.70)** | **1.553** | **0.126** | **1.586** | **1.296 – 1.700** |
|  | **U(0.75,1.50)** | **1.395** | **0.086** | **1.418** | **1.220 – 1.500** |
|  | **U(0.01,0.30)** | **0.254** | **0.033** | **0.261** | **0.190 – 0.300** |
|  | **U(0.40,0.70)** | **0.583** | **0.073** | **0.594** | **0.448 – 0.699** |
|  | **U(0.10,0.90)** | **0.682** | **0.151** | **0.708** | **0.390 – 0.900** |
|  | **U(0.10,0.90)** | **0.748** | **0.113** | **0.774** | **0.524 – 0.900** |
|  | **U(0.50,0.95)** | **0.654** | **0.108** | **0.633** | **0.500 – 0.865** |
|  | **U(0.50,0.95)** | **0.674** | **0.116** | **0.653** | **0.500 – 0.893** |
|  | **U(0.01,0.33)** | **0.116** | **0.083** | **0.091** | **0.010 – 0.285** |
|  | **U(0.01,0.33)** | **0.162** | **0.061** | **0.154** | **0.054 – 0.285** |

**Model SIRS_3_**

| **Parameter** | **Prior distribution (chosen based on literature)** | **Posterior distribution** | | | |
| --- | --- | --- | --- | --- | --- |
|  |  | **mean** | **SD** | **median** | **HPD interval (95%)** |
|  | **U(0.10-1.00)** | **0.593** | **0.207** | **0.586** | **0.246 – 0.968** |
|  | **U(0.10,1.00)** | **0.794** | **0.137** | **0.817** | **0.535 – 1.000** |
|  | **U(0.60,1.70)** | **1.346** | **0.243** | **1.392** | **0.883 – 1.700** |
|  | **U(0.75,1.50)** | **1.339** | **0.123** | **1.367** | **1.097 – 1.500** |
|  | **U(0.01,0.30)** | **0.166** | **0.053** | **0.159** | **0.075 – 0.273** |
|  | **U(0.40,0.70)** | **0.487** | **0.066** | **0.471** | **0.400 – 0.624** |
|  | **U(0.10,0.90)** | **0.453** | **0.207** | **0.438** | **0.100 – 0.813** |
|  | **U(0.10,0.90)** | **0.502** | **0.200** | **0.502** | **0.158 – 0.869** |
|  | **U(0.01,1.00)** | **0.651** | **0.215** | **0.675** | **0.259 – 0.999** |
|  | **U(0.01,1.00)** | **0.693** | **0.195** | **0.719** | **0.332 – 0.999** |

**Model SIRS_4_**

| **Parameter** | **Prior distribution (chosen based on literature)** | **Posterior distribution** | | | |
| --- | --- | --- | --- | --- | --- |
|  |  | **mean** | **SD** | **median** | **HPD interval (95%)** |
|  | **U(0.10-1.00)** | **0.679** | **0.182** | **0.695** | **0.362 – 0.999** |
|  | **U(0.10,1.00)** | **0.862** | **0.102** | **0.885** | **0.660 – 0.999** |
|  | **U(0.60,1.70)** | **1.436** | **0.197** | **1.480** | **1.046 – 1.700** |
|  | **U(0.75,1.50)** | **1.340** | **0.122** | **1.367** | **1.098 – 1.500** |
|  | **U(0.01,0.30)** | **0.172** | **0.052** | **0.167** | **0.085 – 0.276** |
|  | **U(0.40,0.70)** | **0.523** | **0.078** | **0.513** | **0.400 – 0.664** |
|  | **U(0.10,0.90)** | **0.478** | **0.204** | **0.473** | **0.100 – 0.823** |
|  | **U(0.10,0.90)** | **0.656** | **0.160** | **0.681** | **0.354 – 0.900** |
|  | **U(0.01,1.00)** | **0.716** | **0.185** | **0.745** | **0.365 – 0.999** |
|  | **U(0.01,1.00)** | **0.732** | **0.176** | **0.762** | **0.403 – 1.000** |
|  | **U(0.01,0.10)** | **0.033** | **0.017** | **0.030** | **0.010 – 0.069** |
|  | **U(0.01,0.50)** | **0.083** | **0.032** | **0.079** | **0.022 – 0.147** |
